# HAb18G/CD147 cell-cell contacts confer resistance of a HEK293 subpopulation to anoikis in an E-cadherin-dependent manner

**DOI:** 10.1186/1471-2121-11-27

**Published:** 2010-04-17

**Authors:** Xiao-Kui Ma, Li Wang, Yu Li, Xiang-Ming Yang, Pu Zhao, HaoTang, Ping Zhu, Ling Li, Zhi-Nan Chen

**Affiliations:** 1 Cell Engineering Research Center & Department of Cell Biology, National Key Discipline of Cell Biology, State Key Laboratory of Cancer Biology, Fourth Military Medical University, 17 Changle West Road, Xi'an 710032, PR China; 2 Department of Clinical Immunology in Xijing Hospital, Fourth Military Medical University,17 Changle West Road, Xi'an 710032, PR China

## Abstract

**Background:**

Acquisition of resistance to "anoikis" facilitates the survival of cells under independent matrix-deficient conditions, such as cells in tumor progression and the production of suspension culture cells for biomedical engineering. There is evidence suggesting that CD147, an adhesion molecule associated with survival of cells in tumor metastasis and cell-cell contacts, plays an important role in resistance to anoikis. However, information regarding the functions of CD147 in mediating cell-cell contacts and anoikis-resistance remains limited and even self-contradictory.

**Results:**

An anoikis-resistant clone (HEK293ar), derived from anoikis-sensitive parental Human Embryonic Kidney 293 cells, survived anoikis by the formation of cell-cell contacts. The expression of HAb18G/CD147 (a member of the CD147 family) was upregulated and the protein was located at cell-cell junctions. Upregulation of HAb18G/CD147 in suspended HEK293ar cells suppressed anoikis by mediating the formation of cell-cell adhesions. Anoikis resistance in HEK293ar cells also required E-cadherin-mediated cell-cell contacts. Knock-down of HAb18G/CD147 and E-cadherin inhibited cell-cell contacts formation and increased anoikis sensitivity respectively. When HAb18G/CD147 was downregulated, E-cadherin expression in HEK293ar cells was significantly suppressed; however, knockdown of E-cadherin by E-cadherin siRNA or blocking of E-cadherin binding activity with a specific antibody and EDTA had no significant effect on HAb18G/CD147 expression. Finally, pretreatment with LY294002, a phosphoinositide 3-kinase (PI3K/AKT) inhibitor, disrupted cell-cell contacts and decreased cell number, but this was not the case in cells treated with the extracellular signal-regulated kinase (ERK) inhibitor PD98059.

**Conclusions:**

Our results provide new evidence that HAb18G/CD147-mediated cell-cell contact confers anoikis resistance in an E-cadherin-dependent manner; and cell-cell contact mediated resistance to anoikis implicates PI3K pathway in a highly relevant cell model (HEK293ar). Understanding of the role of HAb18G/CD147 cell-cell contacts in anoikis resistance may help in understanding the survival of cells in anchorage-independent growth, such as cells in tumor metastasis and suspension culture produced for biomedical engineering. Our results also contribute to a better understanding of the biology of HEK293 cell spheroids, a major workhorse for producing human therapeutic agents and viral vaccines.

## Background

CD147, an extracellular matrix metalloproteinase inducer (also known as EMMPRIN, basigin, M6), is a plasma membrane-bound glycoprotein that functions as an adhesion molecule. It is expressed at high levels on a variety of malignant human cancers and some immortalized cell lines. Our laboratory previously identified a novel hepatoma associated antigen named HAb18G, which was obtained by cloning a human hepato-cellular carcinoma (HCC) cDNA library and screening with the anti-hepatoma monoclonal antibody HAb18 [1]. The nucleotide acid and amino acid sequences of HAb18G are identical to those of CD147 [2]. HAb18G/CD147 was highly expressed by HCC cells and tissues, and increased HAb18G/CD147 expression stimulated both the growth and invasiveness of HCC cells, much as CD147 functions in other cancer cells [3-5].

The acquisition of resistance to anoikis, a form of apoptosis triggered by loss or alteration of cell-cell or cell-matrix anchorage, is critical for the survival of cells in tumor progression and suspension growth used in engineering. Resistance to anoikis is emerging as a hallmark of metastatic cancer cells, important in tumor progression because it increases survival times in the absence of cell anchorage, facilitating migration and reattachment, and therefore increasing the probability of metastasis [6]. Furthermore, acquisition of anoikis resistance is required for cells used in engineering during adaptation to suspension culture and spheroid growth.

More recently, CD147 has been reported as an anoikis suppressor, promoting anchorage-independent growth by stimulating hyaluronan production [7] and regulating the anoikis signal pathway by upregulating Bim [4]. However, it is not clear whether the role of CD147 in anoikis resistance is related to cell adhesion, which is a basic function of this molecule in addition to its role in stimulating matrix metalloproteinase (MMP) secretion [8]. Different bioactive epitopes of CD147 involved in regulating cell adhesion have been identified [9]. CD147 has also been reported to participate in forming compacted cell aggregates by regulating fibronectin matrix assembly [4] and cell-cell adhesion [10]. The binding of CD147 mAb to CD147 may mimic natural ligand-receptor binding and induce homotypic U937 monocytic cell aggregation via the LFA-1/ICAM-1 pathway [11]. In contrast, Cho reported that antibodies to CD147 are potent inhibitors of homotypic U937 aggregation induced via CD98 ligation [12]. Because the establishment/maintenance of cell-cell contacts is considered an important environmental condition for physiological resistance to anoikis, we hypothesized that CD147 may confer anoikis resistance by mediating cell-cell adhesion. Unfortunately, direct evidence for the role of CD147 in mediating cell-cell contacts and anoikis resistance is very limited and even self-contradictory. Furthermore, it is not clear whether CD147 is directly involved in cell adhesion either as an adhesion signal transmitting molecule or a regulator.

The aim here was to explore whether HAb18G/CD147 is involved in forming cell-cell contacts, and whether this contributed to its role in regulating anoikis resistance. A transformed cell line, Human Embryonic Kidney (HEK) 293, was chosen as the model because our previous results confirmed that these cells express HAb18G/CD147 [13]. We also acquired an anoikis-resistant subpopulation (HEK293ar) from the anoikis-sensitive parental HEK293 cells. Together, these two cell types provide an ideal model for exploring the role of HAb18G/CD147 as a cell-cell adhesion molecule preventing anoikis. Our results show that HAb18G/CD147 cell-cell contacts confer anoikis resistance and this function also involves E-cadherin expression in HEK293ar cell spheroids.

## Results

### Anoikis-resistant HEK293ar cells survive anoikis by the formation of cell-cell contacts

**Figure 1:**
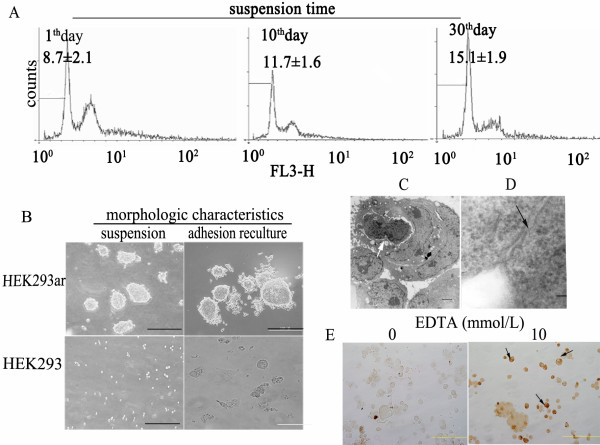
**Anoikis-resistant HEK293ar cells survives anoikis through cell-cell contacts**. An anoikis-resistant HEK293 cell subpopulation (HEK293ar) was obtained by sequential cycles of adhesion and suspension culture. **A**. Anoikis in HEK293ar cells in suspension culture at different time points. Flow cytometry (histogram) shows that the apoptosis rate changed little during suspension culture of HEK293ar. The x and *y*-axes indicate the size of DNA and the number of cells counted, respectively. FL3-H, a standard term for flow cytometry, represents measurement of the fluorescence intensity of propidium iodide (PI) at a super-red wavelength (670 nm). The results are means ± S.D. (n = 4-6). **B**. The morphology of HEK293ar and parental HEK293 cells in suspension and adhesion culture revealed by microscopy (n = 4-10). Magnification: ×200. **C**. TEM of a multicellular HEK293ar spheroid. Chromatin masses of moderate electron density are dispersed in the nuclei and nucleoli are conspicuous (arrowed). The cells were observed in 4-6 independent sections. Bar, 2 μm. **D**. A junctional complex with thickened membranes (arrowed) in suspended HEK293ar cells viewed under SEM. Bar, 50 nm. The cells were viewed in 4-10 independent sections (at least 100 cells/section). **E**. EDTA (10 mmol/l) treatment disrupted the cell-cell contacts and resulted in massive apoptosis as determined by TUNEL assay. Arrowheads show that single cells detached from the aggregates give more intense TUNEL staining. Magnification: ×200. The apoptotic nuclei were counted in 4-10 independent sections (at least 500 nuclei/section).

To explore the role of cell-cell contacts in preventing anoikis, we generated a subpopulation (HEK293ar) with an anoikis-resistant phenotype derived from HEK293 cells using sequential cycles of adhesion and suspension culture [14]. Upon detachment, the apoptotic ratio in HEK293ar cells was lower than in the parental cells, as shown by quantifying the proportions in sub-G1 by flow cytometry (6.97 ± 3.1% versus 37.3 ± 2.3%, data not shown). When HEK293ar cells were cultured in suspension for 1, 10 and 30 days, the mean sub-G1 proportions were 8.7, 11.7, and 15.1%, respectively, with little change in the apoptotic ratio (Fig. 1A). Moreover, this subpopulation maintained the anoikis resistance phenotype in vitro for 6 months (data not shown). Additionally, after ~6-10 h suspension culture, the HEK293ar cells spontaneously and gradually formed cell-cell contacts and generated multi-cellular spheroids that could still grow as adhesion cultures, whereas the parental HEK293 cells did not (Fig. 1B). Ultrastructural examination demonstrated that the suspended spheroids comprised many single cells. In each spheroid, chromatin masses of moderate electron density were dispersed in the nuclei, and the nucleoli were conspicuous (Fig. 1C, arrowed). Cells within the multi-cellular spheroids adhered laterally to each other through diverse specialized intercellular junctions (Fig. 1D, arrowhead). Pretreatment with EDTA solution to disrupt calcium-dependent cell-cell contacts also disrupted the adhesions and caused massive DNA fragmentation in HEK293ar cells, as shown by TUNEL assay (Fig. 1E). Interestingly, single cells detached from the aggregates gave more intense TUNEL staining (Fig. 1E, arrowhead). In contrast, TUNEL assays and ultrastructural examination demonstrated that HEK293 cells clearly underwent anoikis (data not shown). It was previously reported that cell-cell contacts prevent anoikis in primary human colonic epithelial cells [15]. The present results also suggest that adjacent HEK293ar epithelial cells form cell-cell contacts, suppressing anoikis under matrix-deficient conditions.

### HAb18G/CD147 promotes HEK293ar cell survival in suspension by mediating cell-cell contacts

**Figure 2:**
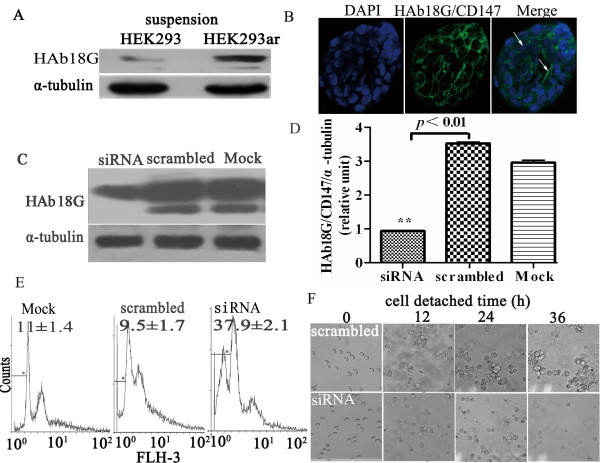
**HAb18G/CD147 promotes HEK293ar cells survival by mediating cell-cell contacts**. **A**. Western blotting to reveal HAb18G/CD147 expression in HEK293ar and parental HEK293 cells in suspension culture. Two major forms of HAb18G/CD147 (43-66 and 35 kDa) were analyzed. α-Tubulin was used as a loading control (n = 4-6). **B**. Immunofluorescence of HAb18G/CD147 under laser scanning confocal microscope (Olympus FV1000, Tokyo, Japan; n = 4-10). The nuclei were counterstained with DAPI (4',6-diamidino-2-phenylindole). Magnification: ×1200. **C**. Western blotting to reveal HAb18G/CD147 expression in HEK293ar cells in HAb18G/CD147 RNA interference (n = 4-6). α-Tubulin was used as a loading control. **D**. Comparison of the gray scale ratio of HAb18G/CD147/α-tubulin in HAb18G/CD147 RNA interference (n = 4-6). ** *p *< 0.01, siRNA versus scrambled. **E**. Flow cytometric quantification of anoikis after siRNA treatment (n = 4-6). Anoikis was determined as described in Fig. 1A. **F. C**ell-cell contacts formation with time after treatment of targeted HAb18G/CD147-siRNA under the phase-contrast microscope (n = 4-6). Magnification: × 400. Each value represents the mean ± SD of at least triplicate determinations. Results are the representative of three similar experiments.

We next explored whether HAb18G/CD147 was involved in HEK293ar cell spheroid survival by mediating cell-cell contacts. HAb18G/CD147 expression was compared between HEK293ar and HEK293 cells in suspension culture. HEK293ar cells expressed significantly more HAb18G/CD147 than the parental cells after 24 h (Fig. 2A). Positive immunofluorescence staining for CD147 was restricted to the cell-cell contacts of HEK293ar (Fig. 2B, merge, arrowhead). These results may imply a correlation between HAb18G/CD147expression and cell-cell contacts-directed anoikis resistance, as the level of HAb18G/CD147 expression was similar in both cell types in adhesion culture (data not shown). To obtain further insight into this correlation, HEK293ar cells were treated with HAb18G/CD147 siRNA in adhesion culture, and apoptosis and cell-cell contacts formation were determined in suspension culture. When HAb18G/CD147 expression was reduced by ~70% (Figs. 2C, D; **p < 0.01), HEK293ar cells underwent anoikis with a sub-G1 proportion of 37.9 ± 2.1% (Fig. 2E). Cell-cell contacts formation was totally inhibited after suspension, whereas the control cells formed multicellular aggregates after as little as 12 h in suspension (Fig. 2F). These results indicate that HAb18G/CD147 confers anoikis resistance of HEK293ar cells to anoikis by mediating cell-cell contacts formation.

### Cell-cell contact-directed survival in suspension involves E-cadherin

**Figure 3:**
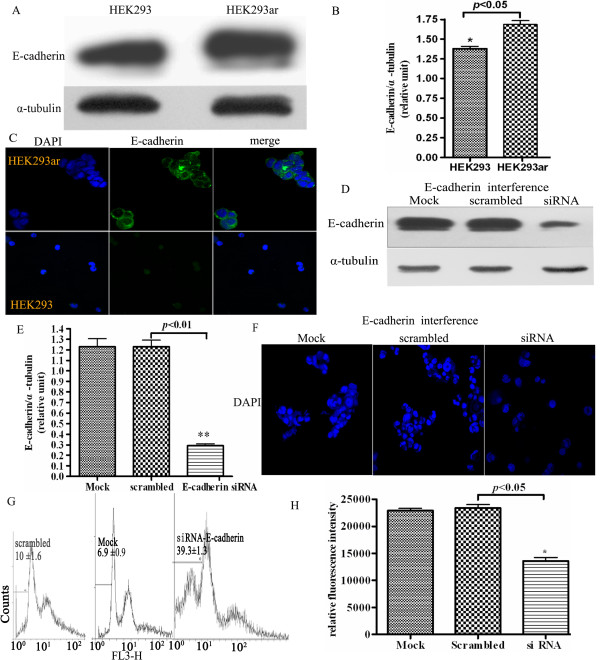
**Cell-cell contact-directed HEK293ar cell survival requires the involvement of E-cadherin**. **A**. Increased E-cadherin expression in HEK293ar cells revealed by western blotting (n = 4-6). **B**. Comparison of the gray scale ratio of E-cadherin/α-tubulin between HEK293 and HEK293ar cells (n = 4-6). * *p *< 0.05, HEK293 versus HEK293ar. **C**. E-cadherin expression in HEK293ar and HEK293 cells revealed by immunofluorescence under laser scanning confocal microscope (Olympus FV1000, Tokyo, Japan; n = 4-10). Magnification: × 1600. **D**. Western blotting to reveal E-cadherin expression in E-cadherin RNA interference (n = 4-6). **E**. Comparison of the gray scale ratio of E-cadherin/α-tubulin in E-cadherin RNA interference (n = 4-6). ** *p *< 0.01, siRNA versus scrambled. **F**. The effect of E-cadherin RNA interference on the cell-cell contacts formation. Magnification: × 1000. The nuclei were counterstained with DAPI. The cells were viewed in 4-6 independent sections (at least 300 cells/section). **G**. Flow cytometric quantification of anoikis after E-cadherin siRNA-treatment (n = 4-6). **H**. The effect of E-cadherein knockdown on the cell number of HEK293ar in suspension. * *p *< 0.05, siRNA versus scrambled. The quantification of relative cell number was done with CyQUANT^® ^NF Cell Proliferation Assay Kit. This kit measures the cellular DNA content via fluorescent dye binding, which is closely proportional to cell number (n = 4-10). Each value represents the mean ± SD of triplicate determinations. Results are the representative of three similar experiments.

Upon detachment, anchorage-independent Ewing sarcoma cells suppressed anoikis through a pathway involving E-cadherin-dependent cell-cell adhesion [16]. Thus we explored whether E-cadherin was also involved in suppressing anoikis by mediating cell-cell adhesion in HEK293ar cells. As anticipated, increased E-cadherin expression was confirmed in HEK293ar cells after 24 h suspension culture (Figs. 3A, B, *p < 0.05), and positive staining was partly located around the cell-cell contacts (Fig. 3C). Also, no significant expression of E-cadherin in completely suspended parental HEK293 cells or in the initially suspended HEK293ar cells was found by immunofluorescence. A reasonable explanation could be that E-cadherin expression decreases dramatically upon cell-cell detachment [17]. In addition, a 70-80% reduction of E-cadherin expression by siRNA (Figs. 3D, E; ** *p *< 0.01), as determined by western blotting, totally inhibited cell-cell contacts formation (Fig. 3F) and decreased the degree of cell aggregation with 70-80%, scored as described in Methods. Knockdown of E-cadherin led to a marked increase in the mean sub-G1 proportion and a little shifted peaks in Flow cytometric histogram (Fig. 3G, * *p *< 0.05), and it also decreased the cellular DNA content via fluorescent dye binding determined with a CyQUANT^® ^NF Cell Proliferation Assay Kit (Fig. 3H; *p < 0.05), which is closely proportional to cell number. These results suggest that cell-cell contacts formation and anoikis resistance in HEK293ar cells may also be mediated by E-cadherin.

### HAb18G/CD147 mediates cell-cell contacts and anoikis resistance through E-cadherin cell-cell contacts

**Figure 4:**
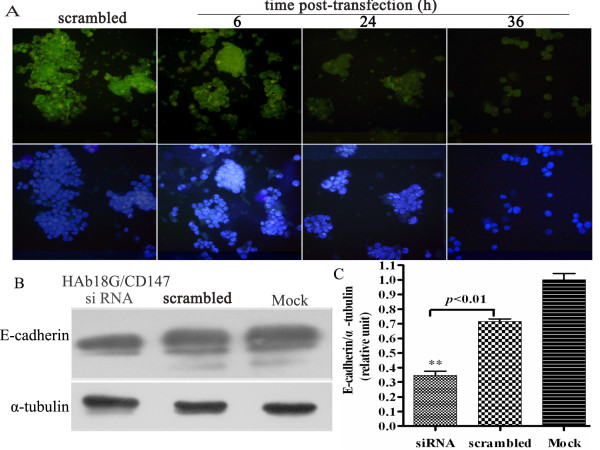
**HAb18G/CD147 knockdown reduces the expression of E-cadherin**. **A**. E-cadherin expression and the formation of cell-cell contacts with time after HAb18G/CD147 siRNA treatment (n = 4-6). Magnification: × 400. **B**. Western blotting to reveal E-cadherin in HAb18G/CD147-siRNA-treated HEK293ar cells in suspension culture (n = 4-6). α-Tubulin was used as a loading control. **C**. Comparison of the gray scale ratio of E-cadherin/α-tubulin in HAb18G/CD147-siRNA treatment (n = 4-6). ** *p *< 0.01, siRNA versus scrambled. Each value represents the mean ± SD of triplicate determinations. Results are the representative of three similar experiments.

**Figure 5:**
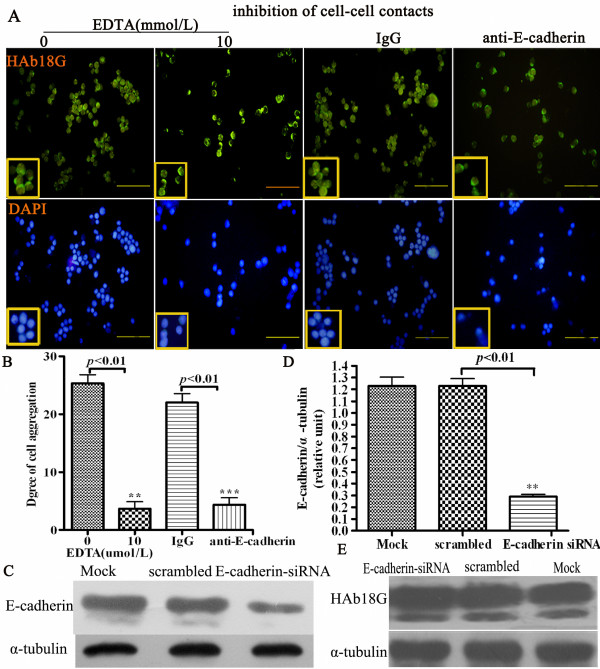
**Effect of E-cadherin blocking or knockdown and EDTA on the cell-cell contact formation and HAb18G/CD147**. **A**. The effect of EDTA and anti-E-cadherin treatment on HAb18G/CD147 expression and cell-cell contacts formation of HEK293ar cells in suspension (n = 4-10). Magnification: ×400. **B**. The quantification of effect of EDTA and anti-E-cadherin treatment on the cell aggregation (n = 4-10). The degree of cell aggregation was scored as described in Methods. The cells were counted in 4-10 independent sections (at least 300 nuclei/section). ** *p *< 0.01, EDTA(0) versus EDTA(10); ***p = 0.008< 0.01, IgG versus anti E-cadherin. **C**. Western blotting to reveal E-cadherin expression in HEK293ar cells in E-cadherin RNA interference (n = 4-6). **D**. Comparison of the gray scale ratio of E-cadherin/α-tubulin in E-cadherin RNA interference (n = 4-6). ** *p *< 0.01, siRNA versus scrambled. **E**. Western blotting of HAb18G/CD147 when E-cadherin was knocked down by E-cadherin siRNA (n = 4-6). *p *= 0.4775, si RNA versus scrambled. Each value represents the mean ± SD of triplicate determinations. Results are the representative of three similar experiments.

As HAb18G/CD147 and E-cadherin are both related to cell-cell contact-directed survival of HEK293ar cells in suspension, they may be functional connected. To test this hypothesis, we investigated the relationship between these two molecules by RNAi. Figs. 4A, B and 4C show that E-cadherin expression decreased dramatically in HAb18G/CD147 siRNA-treated HEK293ar cells according to immunofluorescence staining density (Figs. 4A, analyzed by Image Pro Plus 6.0 3-DS software, **p < 0.01, data not shown) and western blotting (Fig.4C, **p < 0.01). Interestingly, with time after HAb18G/CD147 siRNA treatment, cell-cell contacts and spheroids gradually disrupted (Fig. 4A). In contrast, blocking of E-cadherin binding by an anti-E-cadherin and inhibition of cell-cell contacts with EDTA showed no significant effect on HAb18G/CD147 expression at the single cell level according to immunofluorescence staining (Fig. 5A; fluorescence density was analyzed by Image Pro Plus 6.0 3-DS, **p < 0.01, data not shown), although the degree of cell aggregation was decreased in suspension culture (Fig. 5B, **p < 0.01). Furthermore, treatment of HEK293ar cells with E-cadherin siRNA under adhesion conditions did not alter the level of HAb18G/CD147 expression of HEK293ar in suspension (Figs. 5C, D, ** *p *< 0.01; Fig. 5E, *p *> 0.05). Together, these results indicate that the effect of HAb18G/CD147 on cell-cell adhesion and anoikis suppression is mediated by E-cadherin.

### PI3K pathway is involved in the cell-cell mediated resistance to anoikis

**Figure 6:**
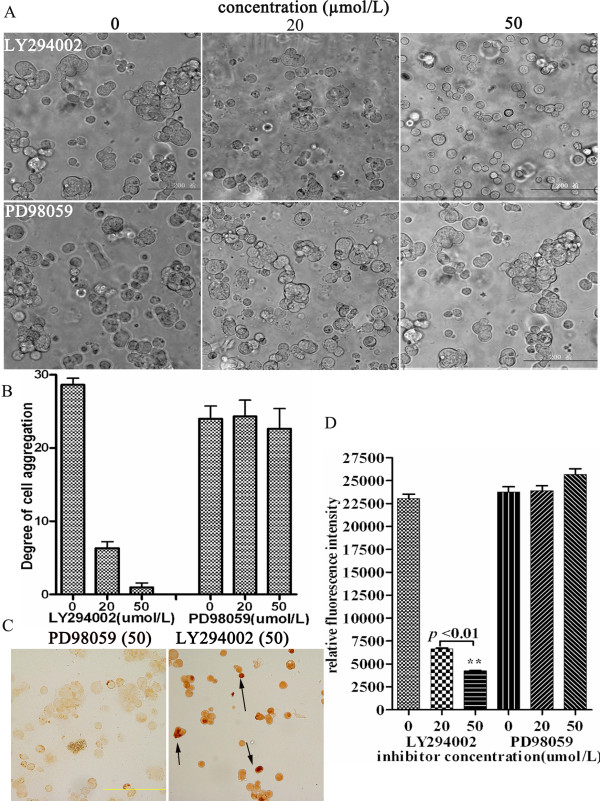
**The suppression of anoikis in HEK293ar mediated by cell-cell contacts involves the PI3K/Akt pathway**. **A**. The effect of the PI3K (phosphoinositide 3-kinase) inhibitor LY294002 (20, 50 μmol/l) or the ERK (extracellular signal-regulated kinase) inhibitor PD98059 (20, 50 μmol/l) on cell-cell contacts formation (n = 4-10). Magnification: × 200. **B**. The effect of signal pathway inhibitors treatment on the degree of cell aggregation (n = 4-10). The cells were counted in 4-10 independent sections (at least 300 nuclei/section). **C**. The effect of signal pathway inhibition on the survival of cells in suspension. The anoikis was determined with TUNEL assay kit. The apoptotic nuclei were counted in 4-10 independent sections (at least 500 nuclei/section). The concentration of both LY294002 (PI3K inhibitor) and PD98059 (ERK inhibitor) is 50 μmol/l. Magnification: ×200. **D**. The effect of signal pathway inhibitors LY294002 (0, 20, 50 μmol/l) or PD98059 (0, 20, 50 μmol/l) on cell number. It was evaluated with a CyQUANT^® ^NF Cell Proliferation Assay Kit (C35006, Invitrogen, Ltd) according to the manufacturer's protocol for the nonadherent cells. ** *p *< 0.01, LY294002 (20) versus LY294002 (50). Newman-Keuls Multiple Comparison Test. Each value represents the mean ± SD of at least triplicate determinations. Results are the representative of three similar experiments.

The Ras-ERK1/2 or PI3K/AKT pathway is involved in E-cadherin or HAb18G/CD147-mediated cell adhesion and survival [18-20]. To determine the situation, we investigated whether these two pathways were involved in cell-cell contact-directed survival in suspension. HEK293ar spheroids were treated with LY294002 (a specific competitive PI3K inhibitor) or PD98059 (an ERK inhibitor). As shown in Figs. 6A, B, LY294002 (20-50 μmol/l) inhibited the cell-cell contacts formation and decreased the degree of cell aggregation in a dose-dependent manner, but not PD98059. TUNEL assay showed that the LY294002 (50 μmol/l) treatment resulted in positive staining of TUNEL in HEK293ar cells (Fig. 6C, arrowhead), but PD98059 (50 μmol/l) did not. Additionally, LY294002 (20-50 μmol/l) decreased relative fluorescent intensity indicating the cellular DNA content in a dose-dependent manner (Newman-Keuls Multiple Comparison Test, **p < 0.01,0 μmol/l versus 20 μmol/l; Fig. 6D) and this result indicated that LY294002 treatment decreased the cell number of HEK293ar in suspension in a dose-dependent manner, as the cellular DNA content via fluorescent dye binding is closely proportional to cell number based on the instruction of the assay kit (described in Methods). In contrast, PD98059 did not significantly affect the relative fluorescent intensity even at 50 μmol/l (Fig. 6D). These results indicate that PI3K pathway inhibition suppresses cell-cell contacts formation and results in decrease of cell number in suspended HEK293ar cells.

## Discussion

Anoikis resistance is a key to the survival of cells in malignant transformation and metastasis [18,19]. It may also be a key to the adaptation of cells to suspension culture and spheroids growth used in engineering. For epithelial cells, suppression of anoikis upon detachment seems to be induced when cell-cell contacts are formed. For example, cadherin-mediated homotypic interactions maintain the survival of human prostate carcinoma DU-145 cells in the absence of extracellular matrix (ECM) attachments [20]. Also, disruption of E-cadherin cell-cell contacts showed more important for suppressing anoikis of normal enterocytes after detachment from villus epithelium, as compared to cell-ECM disruption [17]. Growth as spheroids renders tumor cells less sensitive to exogenous apoptotic stimuli, and spheroids have greater drug resistance than the corresponding monolayers [21]; and some cells used in engineering also grow as spheroids in suspension. Thus, elucidating the mechanisms by which spheroids survive through cell-cell contacts has potentially profound value for understanding survival mechanism of such cells and may also be applied to the control of growth of cells used in biomedical engineering. However, much less is known about how survival pathways are activated under anchorage-independent matrix-deficient conditions in these cells.

Recently, Marieb et al. found that the adhesion molecule CD147 promotes anchorage-independent, hyaluronan-dependent growth of human breast carcinoma cells [7]. CD147 is also reported to regulate cell-matrix adhesion [22]. However, it is not clear whether CD147-mediated cell-cell contact has a role in anoikis resistance or the growth of spheroids of cells used in engineering. Using our sophisticated model, which we established from anoikis-resistant HEK293ar and the parental HEK293 cells, we found that HAb18G/CD147 cell-cell adhesion suppresses anoikis in an E-cadherin-dependent manner. In addition, we have shown that cell-cell adhesion-based survival signals arising from adjacent HEK292ar cells may inhibit anoikis in a PI3K/AKT-dependent manner (Figs. 6A, B, C, D). In all, our results indicate that HAb18G/CD147-mediated cell-cell contacts mediate anoikis resistance specifically through an E-cadherin-dependent pathway, and that anoikis suppression mediated by cell-cell contacts in HEK293ar cells involves the PI3K/Akt pathway.

Although the data suggest that elevated HAb18G/CD147 expression is correlated with the progression and invasion potentials of human hepatoma cells [23,24], the role of HAb18G/CD147-mediated cell-cell contacts in acquiring resistance to anoikis remains obscure. Earlier evidence suggested that CD147 promotes cancer cell survival by regulating intercellular contacts and inhibiting anoikis [4]; in that study, cells transfected with the CD147 gene and expressing different levels of CD147 were used. In our study, we first found evidence that elevated endogenous expression of HAb18G/CD147 contributes to cell-cell adhesion and subsequently confers resistance to anoikis under suspension conditions (Figs. 2A, B, C, E, F). Our anoikis-resistant HEK293ar cell model would be more suitable for investigating cell-cell contact-directed anoikis suppression, since it is closer to the natural in vivo status of physiological models and cells used in engineering. In addition, owing to the use of HEK293 cells in biotechniques, our cell model and the elucidation of the cell spheroid mechanism may be relevant to bioengineering.

Cell-cell contact-triggered cell survival also involves anti-apoptotic signalling through E-cadherin-, Src-, and PI3K/Akt-dependent pathways [15,25]. For instance, E- cadherin, a classical cadherin that promotes calcium-dependent cell-cell adhesion, suppresses anoikis in squamous carcinoma and normal proximal tubular cells [25,26]. In Ewing tumor spheroids, E-cadherin cell-cell contacts may activate the ErbB4 RTK signal pathway [16]. Homophilic E-cadherin binding is also involved in activating Akt kinase, which ultimately inhibits caspase-3 activity and prevents anoikis [27]. We found that E-cadherin expression was elevated in the anoikis-resistant HEK293ar cells (Figs. 3A, B, C) after 24 h suspension culture. Also, high levels of E-cadherin expression inhibited anoikis by mediating cell-cell contacts upon cell-matrix detachment, which was required by HAb18G/CD147, functioning as an anoikis suppressor. Likewise, knockdown of HAb18G/CD147 disrupted cell-cell contacts and anoikis ensued (Figs. 2C, D, E, F). In addition, as comparison of E-cadherin expression between the HEK293ar and its parental cell focused on the contrast of this protein after 24 h suspension culture, this time course should be long enough for the stabilisation of ligated E-cadherin and this may preclude the E-cadherin increase from the stabilisation of ligated E-cadherin at sites of adhesion and therefore help in valuing the function of this protein accurately in anoikis resistance. An important conclusion is that HAb18G/CD147 may have a more prominent role in suppressing anoikis than E-cadherin, and E-cadherin may be a downstream effector of CD147 in the development of anoikis resistance, although the precise mechanisms by which E-cadherin complexes are remodeled and degraded remain to be determined.

The pronounced effect of HAb18G/CD147 knockdown on cell survival may result from a direct decrease in E-cadherin expression and subsequent loss of cell-cell contacts (Figs. 4A, B, C). In addition, our more recent data have shown that LY294002, a specific PI3K inhibitor, significantly reduced the effect of HAb18G/CD147 on cell adhesion and metastatic invasion (*p *< 0.01) of human hepatoma cells [5]. E-cadherin, predominantly expressed at cell-cell contacts, is stably bound to the PI3K complex; this protein expression is necessary and sufficient for activating the PI3K/AKT pathway [28]. We have also demonstrated that HAb18G/CD147-mediated cell-cell contacts can mediate cell survival under anchorage-independent condition in an E-cadherin-dependent manner; and that cell-cell contacts mediated resistance of anoikis involves PI3K pathway in our model. Based on all these results, we may, at least in part, infer that HAb18G/CD147 conferred anoikis resistance through E-cadherin mediated cell-cell contacts, which may activate the PI3K/AKT pathway and promote spheroids formation by establishing cell-cell contacts. However, we do not wish to imply HAb18G/CD147 or E-cadherin stimulates survival via PI3K stimulation, since this does not occur directly in response to CD147 and E-cadherin upregulation in our model. And how HAb18G/CD147 affects E-cadherin expression needs further investigation.

## Conclusions

Using a sophisticated anoikis-resistant model that we have developed reveals a novel role of HAb18G/CD147 in cell-cell adhesion leading to anoikis resistance and cell spheroid growth. Our results support the observation that HAb18G/CD147 confers anoikis resistance through E-cadherin-mediated cell-cell contacts; and that cell-cell mediated resistance of anoikis has been shown to involve the PI3K pathway. As cell- contact-directed survival is important for tumor cells in metastasis and invasion, these results should be relevant to a bitter understanding of the cell survival mechanism seen in tumor progression. Furthermore, with the exception of cells of lymphoid origin, most cultures of mammalian cells used in bioengineering need to be adapted to suspension conditions of growth and also capable of forming spheroids in suspension for survival, these results are also relevant to our understanding of the survival mechanism of such cells.

## Methods

### Cell lines and tissue culture

The HEK293 cell line was obtained from the Cell Bank of the Type Culture Collection of the Chinese Academy of Sciences (Shanghai, China). HEK293ar cells were acquired in our laboratory and stocked in the CHINA CENTER FOR TYPE CULTURE COLLECTION (numbered: CCTCC NO: C200927). Cells were cultured in RPMI 1640 supplemented with 10% heat-inactivated fetal bovine serum (FBS) at 37°C in a humidified atmosphere containing 5% CO_2_. Methocel medium consisted of RPMI 1640 supplemented with 0.8% methocel and 10% FBS. The cell detachment culture was a modification of the suspension culture procedure previously described [29]. Briefly, flasks were coated with 1.5-2.0% sterilized agar and supplemented with RPMI 1640/10% FBS. Every 2-3 days, the culture flask was refed regularly by carefully removing the old medium and adding 10-15 ml fresh methocel medium. All cultures were monitored routinely and to ensure they were free of mycoplasma, fungal and bacterial contamination. All cell lines were discarded after two months and new lines were propagated from frozen stocks.

### siRNA transfection

A small interfering RNA (siRNA) targeting E-cadherin (Genbank accession No. NM_004360) was generated: siRNAE-cad 5'-CAGACAAAGACCAGGACUA-3' [30]. siHAb18G/CD147 (5'-GUUCUUCGUGAGUUCCUCdTdT-3', 3'-dTdTCAAGAGCA CUCAAGGAG-5') [23] and siE-cadherin-cad were synthesized by Ambion, Inc. Cells were transfected with siRNA using Lipofectin 2000 (Invitrogen, Ltd, USA), according to the manufacturer's instructions. Briefly, HEK293ar cells in exponential growth phase were transfected with the corresponding targeted siRNA (100 nmol/l) or a scrambled RNA, or mock-treated with Lipofectin 2000, serum- and antibiotic-free RPMI 1640 medium (control). Transiently transfected cells were grown for 24 h and re-plated on agar-coated six-well plates. The cells were incubated for a further 24 h before the experiments were conducted. Silencer negative control siRNA (Ambion, USA) was used under similar conditions as a negative control.

### Apoptosis assay

The morphology of apoptotic cells was assessed by phase-contrast (Olympus, Japan) and electron microscopy (EM). Briefly, cells were cultured under cell detachment conditions for the indicated times, then observed and photographed. To evaluate the subcellular morphology characteristic of apoptosis, transmission electron microscopy (TEM, JEM-2000EX, JEOL, Japan) and scanning electron microscopy (SEM, S3400 N, HITACHI, Japan) were used, both by a previously-described method [17]. Each sample was viewed in 4-10 independent sections (at least 100 cells/section).

To detect the fragmented DNA of apoptotic cells, a DeadENDEM Colorimetric TUNEL System (Promega, USA) was used, according to the manufacturer's protocol. Cells from detached and attached cultures were scored for incidence of TUNEL (+) staining using phase-contrast microscopy. Apoptotic nuclei (TUNEL) with condensed chromatin were darkly stained.

DNA fragmentation was also quantified by flow cytometry (Becton Dickinson, USA) using a DNA PREP™ Reagents kit (Bechman, Coulter, Inc., USA). Fluorescence intensity of propidium iodide (PI) was measured at 670 nm (FL3-H). In the histogram, the y-axis is the number of cells counted (labelled Counts); the x-axis is the DNA size and content for each cell registered (labelled FL3-H). Cells with normal DNA have more intense fluorescence. Apoptotic cells, which have more fragmented DNA, have lower fluorescence intensity. The sub-G1 region (left side of the normal peak) is apoptotic. The number of cells in the sub-G1 region divided by the total cell count indicates the percentage apoptosis. Each sample with a minimum of 1/10^4 ^cells was examined at least three times. Gating of the side-scatter plot excluded debris and gating of the pulse width plot excluded any remaining cell aggregates from analysis.

### Cell-cell contact s perturbation

Formation of cell-cell contacts was disturbed using a calcium chelator or E-cadherin antibody. Briefly, cells seeded at 2 × 10^5^/ml in agar-coated six-well plates were treated with 0 or 10 mmol/l EDTA for 12 h at 37°C. Alternatively, cells were treated with 10 μg/ml mouse anti-E-cadherin (Abcam Ltd. Cambridge, UK), with the same amount of goat IgG (Sigma, USA) being used as control. The degree of cell aggregation was scored as follows; no aggregation (-), 1-5 cells/aggregate (1+), 6-10 cells/aggregate (2+), 10-15 cells/aggregate (3+), greater than 15 cells/aggregate (4+). Photographs were taken with an Olympus camera under an inverted microscope [11].

### Western blotting

Cells were harvested in a lysis buffer, and total protein quantified using BCA. Equal amounts (20 μg) of protein were subjected to 8% sodium dodecyl sulfate- polyacrylamide gel electrophoresis (SDS-PAGE) under non-reducing conditions. The separated proteins were transferred to polyvinylidene difluoride (PVDF) membranes (Millipore, Bedford, MA), which were immunoblotted with the appropriate primary antibody. The following primary antibodies were used: human hepatoma monoclonal HAb18 (1:5000, prepared in our laboratory) and anti-E-cadherin mouse monoclonal (1:800; Abcam, Ltd. Cambridge, UK). HRP-conjugated rabbit-anti-mouse immunoglobulin (1:5000, Pierce, Rockford, IL) was used as the secondary antibody. α-Tubulin (Abcam Ltd. Cambridge, UK) was used as loading control. As for HAb18G/CD147, two major forms of HAb18G/CD147 (43-66 and 35 kDa) were analyzed as previously described [31]. The immunoreactive bands were visualized using a chemiluminescent substrate detection system (GE, Healthcare, USA). The E-cadherin western blotting was done as previously described [16]. Quantification of bands from two similar experiments was done using Gene Tool Image software (n = 4-6).

### Immunofluorescence

Cells were harvested and dried on coverslips, fixed with ice-acetone for 15 min at 4°C. The fixed cells were blocked with 5% non-fat milk for 1 h before being incubated with HAb18 mAb (1:200) and anti-E-cadherin mouse monoclonal antibody (1:50; Abcam, Ltd. Cambridge, UK) in blocking solution for 2 h and stained with FITC-conjugated anti-mouse IgG (1:1000, Pierce, Rockford) for 1 h. Finally, the nuclei were stained with 100 ng/ml DAPI (4', 6-diamidino-2-phenylindole; Biotium, Hayward, USA) in PBS for 3 min. The stained cells were examined with a laser scanning confocal microscope (Olympus FV1000, Tokyo, Japan). Fluorescence density of the confocal images was measured by Image Pro Plus 6.0 3-DS.

### Treatment with signal transduction inhibitors

HEK293ar cells were treated with the ERK inhibitor PD98059 (0-50 μmol/l; Sigma, USA), the PI3K inhibitor LY294002 (0-50 μmol/l; Sigma, USA), or the DMSO vehicle as a control for 2 h under adhesion conditions. The treated cells were cultured under cell-detachment conditions for the indicated times. Cell-cell contacts were observed with an inverted phase-contrast microscope and cell number was evaluated with a CyQUANT^® ^NF Cell Proliferation Assay Kit (C35006, Invitrogen, Ltd, USA), which is based on measurement of cellular DNA content via fluorescent dye binding. The fluorescence intensity of each sample was measured using a fluorescence microplate reader with excitation at ~485 nm and emission detection at ~530 nm. Experimental protocols of assay were done according to the manufacturer's instructions for the nonadherent cells.

### Statistical analysis

All data were expressed as mean ± SD from at least three independent experiments. Statistical analysis was performed by Student's t-test or Newman-Keuls Multiple Comparison Test, using SPSS 11.5 statistical software. All statistical tests were two-sided and *p *values < 0.05 were considered statistically significant.

## Authors' contributions

XKM and LW carried out the experimental protocols, participated in the design of the experiments, performed experiments, and drafting for manuscript. YL, PZ, HT and XMY collected microscope images of anoikis-resistant cells, and helped draft the manuscript. PZ, LL, and ZNC conceived the study, participated in its design and coordination, and were also involved in drafting the manuscript. All authors read and approved the final manuscript.
